# No genetic adaptation of the Mediterranean keystone shrub *Cistus ladanifer* in response to experimental fire and extreme drought

**DOI:** 10.1371/journal.pone.0199119

**Published:** 2018-06-20

**Authors:** Iván Torres, Antonio Parra, José M. Moreno, Walter Durka

**Affiliations:** 1 Universidad de Castilla-La Mancha. Departamento de Ciencias Ambientales, Toledo, Spain; 2 Helmholtz Centre for Environmental Research, UFZ, Department of Community Ecology, Halle, Germany; 3 German Centre for Integrative Biodiversity Research (iDiv) Halle-Jena-Leipzig, Leipzig, Germany; Northeastern University, UNITED STATES

## Abstract

In Mediterranean ecosystems, climate change is projected to increase fire danger and summer drought, thus reducing post-fire recruitment of obligate seeder species, and possibly affecting the population genetic structure. We performed a genome-wide genetic marker study, using AFLP markers, on individuals from one Central Spain population of the obligate post-fire seeder *Cistus ladanifer* L. that established after experimental fire and survived during four subsequent years under simulated drought implemented with a rainout shelter system. We explored the effects of the treatments on marker diversity, spatial genetic structure and presence of outlier loci suggestive of selection. We found no effect of fire or drought on any of the genetic diversity metrics. Analysis of Molecular Variance showed very low genetic differentiation among treatments. Neither fire nor drought altered the small-scale spatial genetic structure of the population. Only one locus was significantly associated with the fire treatment, but inconsistently across outlier detection methods. Neither fire nor drought are likely to affect the genetic makeup of emerging *C*. *ladanifer*, despite reduced recruitment caused by drought. The lack of genetic change suggests that reduced recruitment is a random, non-selective process with no genome-wide consequences on this keystone, drought- and fire tolerant Mediterranean species.

## Introduction

Under current climate change, many plant populations are expected to undergo strong directional selection pressures [1–4]. Organisms can respond to changing environments via a combination of mechanisms such as range shifts [5,6] or adaptation to the new conditions via evolution, among other [2,4,7,8,9,10]. Examples of evolution as a response to changes in climate show that some of these responses can be swift and take place in just a few generations. For instance, rapid evolution has been found in pitcher plant-dwelling-mosquitoes that respond to shorter day lengths as a result of warming temperatures [11] or in the advancement of flowering time and other phenotypic traits of a *Brassica* sp. in less than a decade as a result of drought [12,13]. Likewise, genetic differentiation of individual loci has been found as a response to drought during recruitment of the Mediterranean shrub *Fumana thymifolia* [14], or after 15 years of simulated climate change in the grassland plants *Festuca ovina* and *Plantago lanceolata* [15].

Although much of the focus has been set on studies documenting genetic responses to changing climate, lack of responses have also been reported, or positive but very slow responses that cannot keep pace with the speed of current and projected change [2,16, 17]. Such is the case of the legume *Chamaecrista fasciculata* in which antagonistic responses constrained adaptation [18], or the annual plant *Brassica juncea*, which was unable to respond to climate change by genetic adaptation [19]. Similarly, *Drosophila birchii* failed to evolve further resistance to desiccation after over 30 generations [20]. These cases are, however, much scarcer in the literature than those reporting evolutionary responses, but their relevance is higher in the context of increased extinction rates due to climate change [21]. Moreover, despite an increasing number of studies reporting evidences for evolutionary responses to climate change, many of the reported responses might rather be phenotypic [22,23], and thus, evolutionary responses to climate change must provide genetic proof [23,24]. Additionally, the most intense, directional selection pressures will affect the populations living close to the rear end of the species distribution that are close to their physiological limits [16]. Core populations that are distant from their physiological limits, where genetic variation should be high, should be able to show adaptive responses to climate change, but this needs to be tested.

Experimental studies under natural conditions imposing selection regimes that simulate climate change conditions are a useful tool to assess the ecological and evolutionary responses of organisms, since they can provide a realistic context that includes interacting evolutionary processes and lead to predictions of what to expect in wild populations [4]. Previous climate change experiments in natural populations have shown rapid changes in the frequency of alleles that are strong candidates for genetic adaptation to increased drought stress [14,15], but examples like these are still scarce. Furthermore, the combination of genetics and ecological experiments in the field requires a previous understanding of the background genetic patterns. Background genetic structure may arise due to, for instance, isolation by distance (IBD) [25], which can mask or interfere with the experimental effects of the experiment, especially since many statistical tests are unable to cope with IBD.

In Mediterranean type ecosystems, climate change is projected to induce longer summer drought periods and higher frequency and severity of drought events [26, 27]. Additionally, fire activity, in particular large and high severity fires, are also projected to increase due to increased fire danger [28–30]. Mediterranean vegetation is resilient to fires by means of a series of fire-adaptive traits [31] that include resprouting and post-fire recruitment from seeds, which can be protected in seed-bearing structures in the canopy or stored in soil seed banks [32, 33]. Since fire involves a selective pressure, genetic divergence can be expected in contrasting fire scenarios even in short time intervals. Indeed, fire-persistence traits such as serotiny and flammability have been found to be heritable and under selection [34–36].

Some abundant post-fire obligate seeder species have seeds with physical dormancy that is broken by fire. Once moisture is available in fall, seedlings emerge in large numbers after fire to exploit the favorable conditions for recruitment in recently burned areas. The seedling emergence and establishment patterns are closely related to post-fire rainfall [37,38]. Additionally, sensitivity of germination to water stress can increase after exposure to fire cues such as a heat shock [39], so germination and emergence after fire can be strongly reduced by drought [37,40,41]. Since the most critical phases in post-fire regeneration of seeder species are the germination and seedling stages, an increased occurrence of drought episodes immediately after fire may affect the regeneration and persistence of obligate seeder species [42]. It is therefore necessary to evaluate whether the combination of climate-change-induced fire and different degrees of subsequent drought convey a strong selection pressure on obligate seeder species, and whether it promotes genetic changes on their populations. Such changes can include both, effects due to the neutral process of genetic drift during population bottlenecks in the seedling stage, potentially resulting in genome-wide reduced levels of genetic variation, genetic differentiation and spatial genetic structure, and effects due to selection potentially resulting in addition in strong changes of allele frequency at particular loci.

Here we analyze the genetic response of a population of *Cistus ladanifer*, an obligate post-fire seeder, subjected to fire and subsequent drought simulating future climate change, to assess whether post-fire drought is driving genetic changes in the newly recruited population. For this, we performed a genetic marker study on the individuals that had established and survived after four years of drought following burning. We tested whether climate change treatments affected genome-wide levels of genetic variation within and among treatments and whether individual loci were more differentiated than expected, indicative of selection.

## Materials and methods

### Study species

The focus species of this study is the keystone shrub *Cistus ladanifer* L., a perennial plant endemic to the Western Mediterranean region that dominates successional stages on acidic soils after disturbances like woodland clearing or fire. It is an obligate post-fire seeder, and seeds have physical dormancy that is broken by scarification such as mechanical abrasion or exposure to high temperatures [33]. It is an obligate outcrossing species with self-incompatibility that is pollinated by insects [43].

### Study site

**Fig 1:**
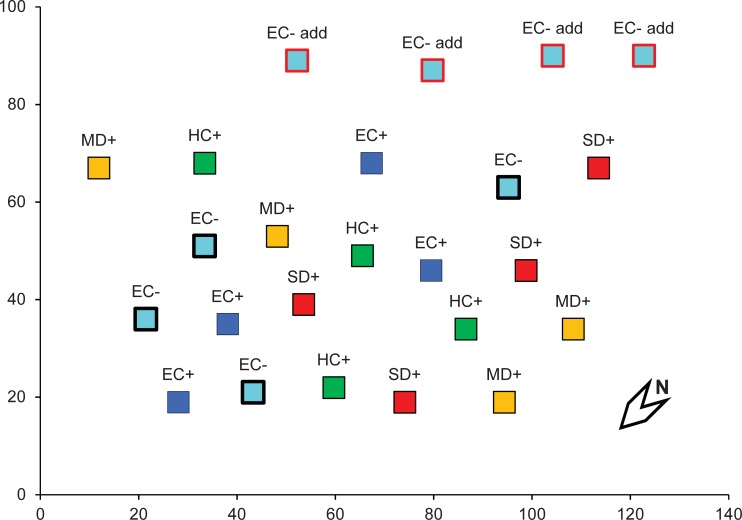
Layout of experimental plots at the Quintos de Mora shrubland. Marks along the axes indicate 20-meter intervals. Symbol labels indicate: EC: Environmental Control, EC add: additional Environmental Control (see text for details), HC: Historical Control, MD: Moderate Drought, SD: Severe Drought; +/-: fire/no fire.

The field study was conducted on public land at the Quintos de Mora Range Station (Los Yébenes, Toledo; 39° 25’ N, 4° 04’ W, elev. 900 m), managed by the Organismo Autónomo Parques Nacionales. The Station staff granted permission for the experiment, and no endangered or protected species were involved. The climate in the area is Mediterranean, with mean annual temperature of 14.9°C and mean annual precipitation of 622 mm (1948–2006, “Los Cortijos” meteorological station; 39° 19’ N, 4° 04’ W). Vegetation consists of a Mediterranean shrubland dominated by *Cistus ladanifer* accompanied by other shrubs like *Erica arborea* L., *Phillyrea angustifolia* L., *Erica scoparia* L. and *Rosmarinus officinalis* L. In a Northwest-facing slope, 20 6 x 6 m plots were selected and a set of experimental rainout shelters were established to manipulate precipitation. The plots were grouped in four blocks perpendicular to the direction of maximum slope of the site, and four treatments of rainfall manipulation were applied: environmental control (EC, natural rainfall, no rainout shelter [501 mm of rain fell on average from 2009 to 2013]), historical control (HC, long-term average rainfall [600 mm], 2 months drought), moderate drought (MD, 25% reduction from long-term rainfall [450 mm], 5 months drought) and severe drought (SD, 45% reduction [325 mm], 7 months drought) (Fig 1). The treatments were applied for 6 months in spring and summer 2009 before burning the plots in September 2009. During the fire, all the plots per block were burned (EC+, HC+, MD+ and SD+), except one EC plot, which was unburned and served as EC- control (Fig 1). All plots burned at high fire intensity, and no significant differences in various fire intensity measures were recorded among rainfall manipulation treatments. After the fire, rainfall manipulation treatments continued for four years. See [44] and [45] for more details on the experimental setup.

### Post-fire plant emergence and survival to drought

None of the *C*. *ladanifer* individuals present in the burned plots survived the fire. The cumulative emergence of new *C*. *ladanifer* plants was much higher in burned treatments (EC+; 170.6 inds. m^-2^) compared to the unburned plots (EC-; 2.0 inds. m^-2^) under natural rainfall. Likewise, the emergence was significantly higher in EC+ or HC+ (101.7 inds. m^-2^) controls than in MD+ and SD+ drought treatments (34.7 and 53.0 inds. m^-2^, respectively) [46]. Four years after fire, plant density was higher in burned (EC+; 18.4 inds. m^-2^) than in unburned plots (EC-; 2.7 inds. m^-2^), and higher in control than in drought treatments (18.4 and 14.4 vs. 3.3 and 3.9 inds. m^-2^ in EC+, HC+, MD+ and SD+, respectively [46]). However, no significant differences in survival between rainfall treatments were recorded, which suggests that final recruitment depends more on the emergence than on the subsequent survival of emerged plants [46].

### Field sampling, AFLP analysis and genotyping

In the spring of the fifth year after the experimental fire, 12 to 20 *C*. *ladanifer* plants per plot were mapped and sampled. To appropriately assess background genetic structure, four additional EC- plots were established in the surroundings of the experimental area (Fig 1). Young, clean, fully grown leaves were collected from each plant. Plant samples were freeze-dried (Telstar Cryodos -50, Spain) and stored in silica-gel until DNA extraction. 12 plant samples per plot were randomly selected for DNA extraction, resulting in 288 individuals analysed. DNA was extracted from 15 mg of ground leaf material with the DNeasy 96 plant kit (Qiagen, Germany) following the indications of the manufacturer, and quantified with a NanoDrop spectrophotometer (Nanodrop Technologies, USA). Genetic variation was analyzed with Amplified Fragment Length Polymorphisms (AFLP, [47]). The protocol was the following: 6μl of each DNA extraction were incubated for 2h at 37°C with 5 u Eco RI (Fermentas Inc.), 1 u Mse I (New England Biolabs Inc.) and 67 u T4 DNA Ligase (BioLabs Inc.), buffered in 0.55 μl BSA, 1.1 μl NaCl [0.5 M], 1.1 μl T4 DNA Ligase buffer (BioLabs Inc.), 50 pmol *Mse* I adapters, and 5 pmol *Eco* RI adapters. 1 μl of this restriction-ligation product was diluted in 4 μl HPLC-grade water. Preamplification of fragments was performed with 4 μl of the restriction-ligation dilution and 16 μl PCR mix that contained 1.5 ng/ μl *Eco*-A and *Mse*-C preselective primers, respectively, 200 μM dNTPs, 0.8 u DreamTaq polymerase (Fermentas Inc.), 2 μl DreamTaq buffer (Fermentas Inc.) and 9.84 μl H_2_O. The thermocycler was programmed for 2 min at 72°C followed by 20 cycles of 20 s at 94°C, 30 s at 56°C and 2 min at 72°C, ending with 30 min at 60°C. 4 μl of the products of preselective amplification were diluted in 36 μl HPLC-grade water. For selective amplification, 2.2 μl of this dilution were combined with 7.8 μl of a PCR mix containing 1.4 μl of forward and reverse fluorescently labelled primers each and 5 μl Multiplex PCR Master Mix (Qiagen). The PCR thermocycler program consisted of 15 min at 95°C and 10 cycles of 20 s at 94°C, 30 s at 66°C (decreasing 1°C per cycle) and 2 min at 72°C, followed by 20 cycles of 20 s at 94°C, 30 s at 56°C and 2 min at 72°C, ending with 30 min at 60°C.

Prior to analysis, a screening of 44 combinations of selective primers was used on 16 randomly selected individuals to find a combination of primers that produced reliable peak patterns. The eight primer combinations selected were FAM-*Eco-*AAC/*Mse-*CTG, VIC-Eco-ACG/Mse-CAA, NED-Eco-ACC/Mse-CTC, PET-Eco-AGG/Mse-CAT, FAM-Eco-ACT/Mse-CACC, VIC-Eco-ACG/Mse-CAT, NED-Eco-ACC/Mse-CAT, and PET-Eco-AGG/Mse-CTAA. Fragment analysis of PCR products was performed on a capillary sequencer ABI 3130 genetic analyzer (Applied Biosystems, USA) with GeneScan LIZ 500 (Applied Biosystems) as internal size standard.

Genotyping of AFLP markers was done in GeneMapper v5.0 (Applied Biosystems) on a total of 282 individuals (AFLP produced inconsistent results in 6 individuals, which were not used further). This produced slightly different sample sizes per plot, but since a restricted number of individuals to obtain similar sample sizes produced quantitatively similar results (see data analysis) we used all data available. We defined loci manually in the range of 50–500 bp and exported peak height data for a total of 526 loci. We then manually adjusted peak height threshold to obtain a presence/absence matrix of each individual allele in each sample. Loci with frequency larger than 0.99 or lower than 0.01, and those with error rates greater than 5% were excluded, resulting in 275 loci with a mean error rate of 2.1%. Error rate was assessed by duplicate genotyping of 32 individuals (11%) from the original DNA extraction, and calculated as the proportion of mismatches between repeated samples over the number of repeated samples. The data resulting from this process is provided in S1 Dataset.

### Data analysis

The background genetic patterns at the study site prior to the experiment were explored by searching for pre-existing spatial genetic structure in the unburned plots (EC-, including additional plots). *C*. *ladanifer* is perennial and thus plants selected in EC- plots were already present at the beginning of the experiment. A pairwise (individual by individual) matrix of Euclidean genetic distance was calculated, and then the original EC- and additional EC- sets of plots were compared with hierarchical Analysis of Molecular Variance (AMOVA) in GenAlEx 6.5 [48,49], using the original/additional sets of plots as regions and the plots as populations. A spatial autocorrelation analysis at the individual level was also performed by comparing spatial and genetic distances of samples from all EC- plots with a correlogram [50] as implemented in GenAlEx. Spatial distance was expressed as the Euclidean distance between plants, based on their geographic coordinates. The autocorrelation coefficient r was calculated for distance classes 0, 0.75, 2, 5, 20, 40, 60 and 100, and significance was assessed with 9,999 permutations. Additionally, a Mantel Test comparing the average genetic and geographic distances per plot was performed in GenAlEx to test for isolation by distance (IBD) at the population level. Significance was assessed with 9,999 permutations.

To analyze the effects of the fire and drought treatments, first the genetic diversity values of each plot were calculated as the mean number of different alleles, number of effective alleles, Shannon’s information index, Genetic diversity (expected heterozygosity, *H*_*e*_, assuming Hardy-Weinberg-equilibrium) and unbiased expected heterozygosity (*uH*_*e*_). Genetic differentiation among plots and treatments was first explored with a Principal Coordinates Analysis (PCoA, covariance-standardized), and then analyzed by means of hierarchical AMOVA with treatment (including EC- additional) as a grouping factor for plots. Variation among plots (Ф_PT_) (overall and pairwise between plots), among treatments (Ф_RT_), and among plots within treatments (Ф_PR_) were calculated as measures of differentiation and tested with 9,999 permutations. Overall Ф_RT_ was also calculated for individual loci, and pairwise tested between plots for those loci significant in the overall test at p<0.01. Several additional, hierarchical AMOVAs were performed to further explore the effect of fire and of drought treatments: EC- vs. EC+, EC+ vs MD+ and SD+, HC+ vs. MD+ and SD+, and between combined fire and no-fire treatments. AMOVAs were performed with slightly different sample sizes for each plot due to unsuccessful amplification of some individuals (see above). Deletion of random individuals to obtain identical sample sizes confirmed qualitatively similar results. Finally, we tested whether fire induced changes in the local genetic spatial structure of *C*. *ladanifer* by running spatial autocorrelation analyses at the within-plot scale (distances <5m) separately for burned and unburned plots. Differences in spatial genetic structure were tested with the heterogeneity test of Smouse et al. [51] and significance was assessed at a p-value of 0.01 after 9,999 bootstraps [52]. Additionally, SGS was quantified with the Sp statistic based on the kinship coefficient [53]. Genetic diversity calculations, AMOVA, PCoA and spatial autocorrelation analyses were run in GenAlEx 6.5, the Sp statistic was calculated with SPAGeDi v. 1.5 [54].

We searched for outlier loci, potentially influenced by selection among treatments, with several different approaches. First, we used a Bayesian approach by running BayeScan 2.1 [55] with a burn-in of 50,000 iterations, a sample size of 5,000 and a thinning interval of 10, which resulted in 100,000 iterations. Additionally, 20 pilot runs of 5000 iterations were run. The value of prior odds for neutral model was set to 10. A locus was considered an outlier at a q-value (the false discovery rate analogue of the p-value) lower than 0.10. This was done for the different treatments as well as for the combined burned and unburned plots to test for the effect of fire. Second, we used the outlier detection method DFDIST/FDIST implemented in the workbench MCHEZA [56] with 100,000 simulations, using the Neutral mean F_ST_ and the Force mean F_ST_ algorithms and setting a false discovery rate of 0.1 and a confidence interval of 0.99. Both Bayescan and Mcheza analyses were run globally for all treatments, and pairwise between specific fire and drought treatments: EC- vs EC+, EC+ vs MD+ and SD+, HC+ vs. MD+ and SD+, and between combined fire and no-fire treatments. Finally, we followed a logistic regression approach by using the software Samβada v0.5.1 [57, 58] (http://lasig.epfl.ch/sambada) to detect the signature of the fire treatment and the drought treatment, expressed as average precipitation of each treatment, on the allele frequencies. A locus was considered an outlier when the G and Wald scores were significant, with Bonferroni correction at a 99% confidence level.

## Results

### Background genetic patterns

**Fig 2:**
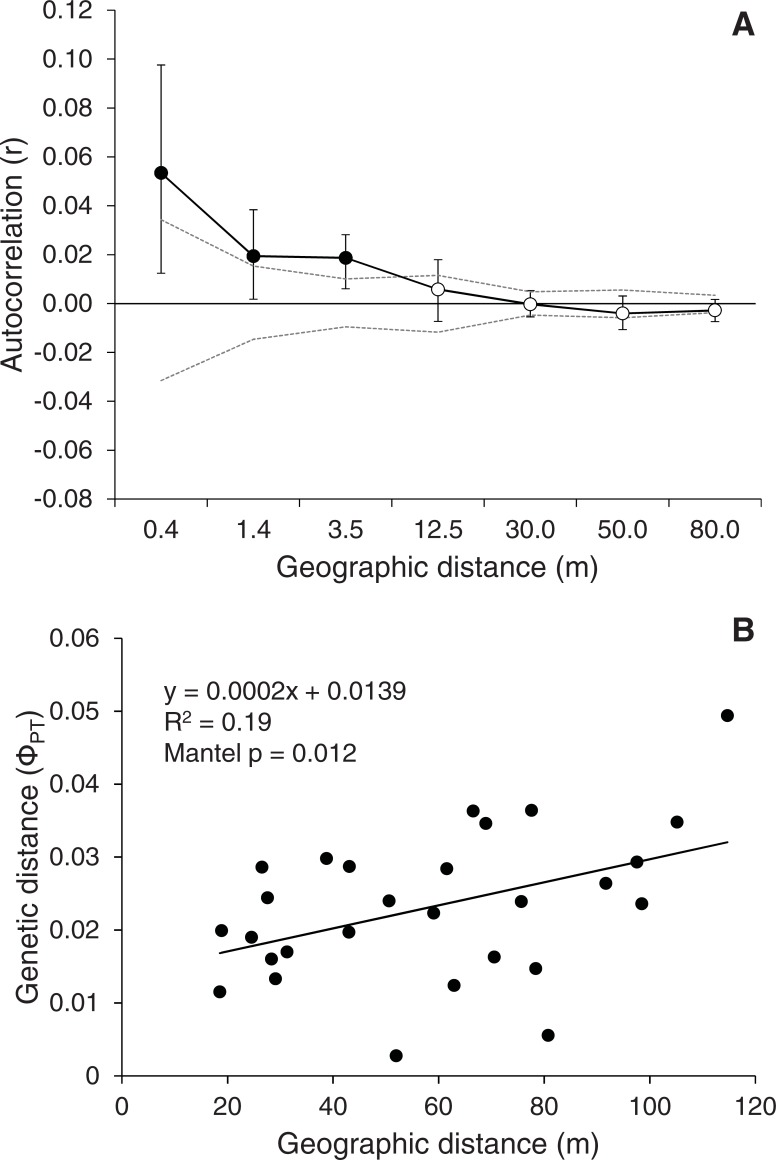
Spatial genetic analysis for *Cistus ladanifer* at the unburned plots (EC-, EC- add). A) Spatial autocorrelogram showing mean r-values per distance class. Filled symbols indicate significant spatial autocorrelation. Dashed lines represent 95% confidence intervals as determined by 9,999 permutations; symbols outside of confidence intervals indicate significance. Error bars depict the 95% confidence interval as determined by 1,000 bootstrap resampling. B) Genetic distance, expressed as pairwise Ф_PT_ among plots, vs. geographic distance.

Analysis of Molecular Variance comparing the original vs. additional unburned plots showed that there was significant genetic structure at the site prior to the onset of the experiment (Ф_RT_ = 0.008, P = 0.008, between additional and original EC- plots). However, genetic variation among plots within these groups (1.9%) was larger than between groups of plots (0.8%) (Table 1). Spatial autocorrelation analysis showed that there was significant spatial genetic structure at the site prior to the experiment (Fig 2A). However, spatial autocorrelation was significant only at distances shorter than 12.5 m and became negative, although not significant, at distances larger than 50 m suggesting a tendency towards more differentiation at larger distances. This is corroborated by a mantel test that showed overall a significant (Mantel p = 0.012) increase of genetic distance among control plots with geographic distance, suggesting a pattern of Isolation By Distance (IBD) (Fig 2B). Overall, the amount of spatial genetic structure was low (Sp statistic = 0.007).

**Table 1:** Hierarchical Analysis of Molecular Variance (AMOVA) showing the partitioning of genetic variation among treatments and plots of *Cistus ladanifer*.

Groups	Variance components	df	Var	% of total	Ф statistics		P
EC- original vs EC- additional							
	Among treatments	1	0.206	0.82	Ф_RT_	0.008	0.008
	Among plots within treatments	6	0.475	1.90	Ф_PR_	0.019	<0.001
	Whithin plots	85	24.306	97.27	Ф_PT_	0.027	<0.001
All treatments							
	Among treatments	5	0.069	0.28	Ф_RT_	0.003	0.023
	Among plots within treatments	18	0.633	2.58	Ф_PR_	0.026	<0.001
	Whithin plots	258	23.823	97.14	Ф_PT_	0.029	<0.001
Burned vs Unburned							
	Among treatments	1	0.023	0.09	Ф_RT_	0.001	0.174
	Among plots within treatments	22	0.682	2.78	Ф_PR_	0.028	<0.001
	Whithin plots	258	23.823	97.12	Ф_PT_	0.029	<0.001
EC- vs EC+							
	Among treatments	1	0.139	0.56	Ф_RT_	0.006	0.044
	Among plots within treatments	6	0.727	2.91	Ф_PR_	0.029	<0.001
	Whithin plots	85	24.103	96.53	Ф_PT_	0.035	<0.001
EC+ vs MD+ and SD+							
	Among treatments	1	0.079	0.33	Ф_RT_	0.003	0.068
	Among plots within treatments	10	0.774	3.20	Ф_PR_	0.032	<0.001
	Whithin plots	130	23.336	96.47	Ф_PT_	0.035	<0.001
EC+ vs MD+							
	Among treatments	1	0.111	0.45	Ф_RT_	0.005	0.065
	Among plots within treatments	6	0.833	3.40	Ф_PR_	0.034	<0.001
	Whithin plots	88	23.557	96.15	Ф_PT_	0.039	<0.001
EC+ vs SD+							
	Among treatments	1	0.000	0.00	Ф_RT_	-0.001	0.557
	Among plots within treatments	6	0.821	3.41	Ф_PR_	0.034	<0.001
	Whithin plots	86	23.262	96.59	Ф_PT_	0.034	<0.001
HC+ vs MD+ and SD+							
	Among treatments	1	0.056	0.23	Ф_RT_	0.002	0.135
	Among plots within treatments	10	0.630	2.60	Ф_PR_	0.026	<0.001
	Whithin plots	129	23.569	97.17	Ф_PT_	0.028	<0.001
HC+ vs MD+							
	Among treatments	1	0.151	0.61	Ф_RT_	0.006	0.020
	Among plots within treatments	6	0.600	2.43	Ф_PR_	0.024	<0.001
	Whithin plots	87	23.904	96.95	Ф_PT_	0.030	<0.001
HC+ vs SD+							
	Among treatments	1	0.000	0.00	Ф_RT_	-0.002	0.726
	Among plots within treatments	6	0.583	2.41	Ф_PR_	0.024	<0.001
** **	Whithin plots	85	23.614	97.59	Ф_PT_	0.022	<0.001

P-value based on 9,999 permutations. Treatment names indicate: +/-: fire/no fire; EC: Environmental Control, EC add: additional Environmental Control (see text for details), HC: Historical Control, MD: Moderate Drought, SD: Severe Drought.

### Treatment effects

Overall genetic diversity of *C*. *ladanifer* was moderate, with mean *H*_*e*_ of 0.127, ranging from 0.119 to 0.135 within plots (Table 2). The average number of effective alleles was 1.196 and was very similar among plots (Table 2), as well as the Shannon Information index (mean 0.206). There were no significant differences for any of the diversity values (*N*_*a*_, *N*_*e*_, *I*, *H*_*e*_, *uH*_*e*_) when all six treatments were compared via one-way ANOVA (S1 Table).

**Table 2:** Genetic diversity metrics of *Cistus ladanifer* under different fire and drought treatments.

Plot	N	*N*_*a*_	*N*_*e*_	*I*	*H*_*e*_	*uH*_*e*_
EC-1	11	1.062	1.198	0.203	0.127	0.133
EC-2	11	1.109	1.208	0.213	0.133	0.139
EC-3	11	1.16	1.202	0.211	0.13	0.137
EC-4	12	1.196	1.202	0.215	0.132	0.137
EC- add1	12	1.171	1.197	0.208	0.128	0.133
EC- add 2	12	1.16	1.195	0.206	0.127	0.132
EC- add 3	12	1.182	1.205	0.214	0.132	0.138
EC- add 4	12	1.102	1.19	0.198	0.122	0.128
EC+1	12	1.095	1.182	0.194	0.119	0.124
EC+2	12	1.182	1.182	0.199	0.12	0.125
EC+3	12	1.167	1.205	0.209	0.13	0.135
EC+4	12	1.164	1.204	0.213	0.131	0.137
HC+1	11	1.12	1.201	0.208	0.129	0.135
HC+2	12	1.178	1.181	0.199	0.12	0.125
HC+3	12	1.225	1.207	0.22	0.135	0.141
HC+4	12	1.2	1.193	0.207	0.126	0.131
MD+1	12	1.149	1.185	0.2	0.122	0.127
MD+2	12	1.098	1.19	0.196	0.121	0.127
MD+3	12	1.233	1.196	0.211	0.128	0.134
MD+4	12	1.153	1.186	0.201	0.123	0.128
SD+1	11	1.153	1.209	0.217	0.134	0.141
SD+2	12	1.135	1.194	0.204	0.126	0.131
SD+3	11	1.022	1.204	0.202	0.127	0.134
SD+4	12	1.055	1.188	0.192	0.119	0.124
Mean	11.750	1.145	1.196	0.206	0.127	0.132

N: Number of individuals, *N*_*a*_: Number of different alleles, *N*_*e*_: Number of effective alleles, *I*: Shannon’s information index, *H*_*e*_: Expected heterozygosity, *uH*_*e*_: Unbiased expected heterozygosity. Plot names indicate: +/-: fire/no fire; EC: Environmental Control, EC add: additional Environmental Control (see text for details), HC: Historical Control, MD: Moderate Drought, SD: Severe Drought.

**Fig 3:**
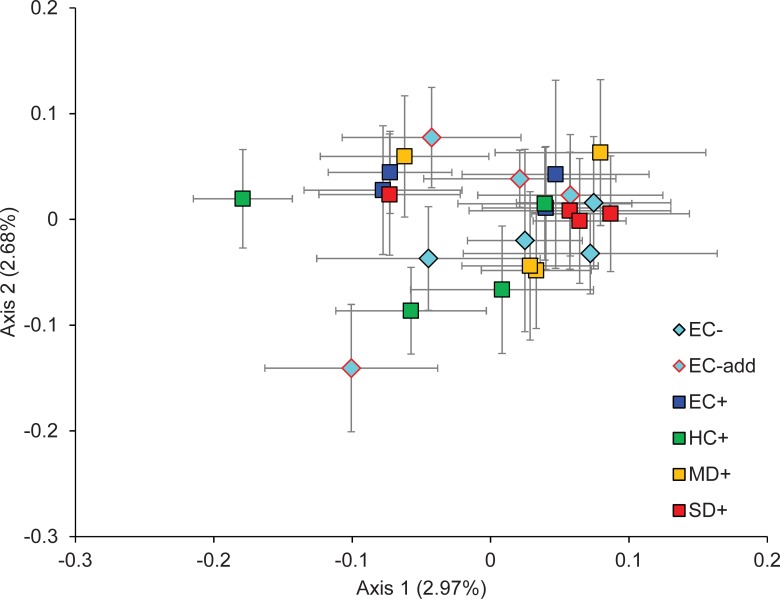
Principal Coordinates Analysis (PCoA) of 24 plots containing 282 individuals of *Cistus ladanifer* subjected to different treatments of fire and drought, based on 275 AFLP loci. EC: Environmental Control, EC add: additional Environmental Control (see text for details), HC: Historical Control, MD: Moderate Drought, SD: Severe Drought; +/-: fire/no fire. Symbols represent the mean position of all individuals within each plot and error bars represent standard error. Cumulative genetic variation explained by the two depicted axes is 5.66%.

**Fig 4:**
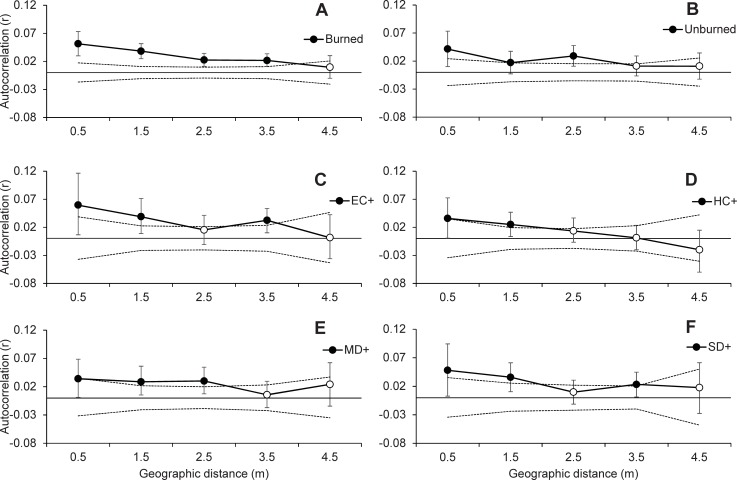
**Local Spatial Genetic Structure of *Cistus ladanifer* in A) burned combined, B) unburned combined, C-F burned and drought plots at the within-plot scale.** EC+: Environmental Control, HC+: Historical Control, MD+: Moderate Drought, SD+: Severe Drought (see text for details). Symbols indicate mean r-values per distance class, filled symbols indicate significant spatial autocorrelation. Dashed lines represent 95% confidence intervals as determined by 9,999 permutations; symbols outside of confidence intervals indicate significance. Error bars depict the 95% confidence interval about r as determined by 10,000 bootstrap resampling.

Analysis of Molecular Variance showed that there was very low but significant genetic differentiation among treatments (Ф_RT_ = 0.003, p = 0.023), which accounted for only 0.28% of genetic variation. Differentiation among plots within treatments was larger (Ф_PT_ = 0.029, p<0.001) and accounted for 2.58% of genetic variation, while the remaining 97.14% of genetic variation occurred within plots (Table 1). The AMOVAs including two treatments showed significant fire effects between EC- and EC+ (Ф_RT_ = 0.006, p = 0.044, 0.56% of genetic variation) and significant drought effects between HC+ and MD+ (Ф_RT_ = 0.006, p = 0.02, 0.61% of genetic variation). The rest of comparisons among treatments showed no significant differences (Table 1). Locus-level AMOVA including all treatments also showed significant (p<0.01) differentiation for the individual loci ACC-CTC_109 and AGG-CAT_213. Locus level AMOVAs including only two treatments showed that these differences tended to occur mostly between plot SD+2 and the other treatments for the case of AGG-CAT_213, while no differences among treatments were found for ACC-CTC_109 (S2 and S3 Tables). PCoA was consistent with the overall AMOVA result and showed a large amount of overlap among treatments (Fig 3). The first two axes captured only a small amount of genetic variation (2.97% and 2.68% for the first and second axes respectively, 5.66% cumulative). Finally, we found significant spatial structure at the intra-plot level (omega = 78.14, p<0.001), but there were no differences in spatial genetic structure between either burned and unburned treatments (omega = 8.301, p = 0.603) or among control and drought treatments (omega = 5.615, p = 0.848) (Fig 4).

Genome scan analyses revealed only few outlier loci, which were inconsistent among methods. Bayescan found no loci potentially under selection at a q-value threshold of 0.10 for any of the treatment comparisons made. The locus with the lowest q-value and highest F_ST_ (0.45 and 0.032, respectively) was ACT-CACC_307 when all treatments were compared. DFDIST/FDIST did not detect any outlier loci when all treatments were analyzed together, but it did find several significant outlier loci when comparing individual treatments: four between EC- and EC+, two between EC+ and SD+, one between HC+ and MD+ and five between burned and unburned treatments (Table 3). Samβada detected only one locus, ACT-CACC_307 (G score = 23.05, Wald score = 21.82), as significantly associated with the fire treatment, while it detected no loci associated with the drought treatments.

**Table 3:** List of potentially adaptive loci and their F_ST_ as identified by the DFDIST/FDIST genome scan approach in pairwise comparisons of drought and fire treatments in *Cistus ladanifer*.

Treatments	loci	F_ST_
EC- vs. EC+	AAC-CTG_232	0.010
	AAC-CTG_262	0.021
	ACG-CAA_464	0.010
	ACC-CAT_199	0.010
EC+ vs. MD+	-	-
EC+ vs. SD+	ACG-CAA_199	0.126
	ACG-CAT_067	0.156
HC+ vs. MD+	ACG-CAA_139	0.123
HC+ vs. SD+	-	-
Burned vs. Unburned	AAC-CTG_122	0.010
	ACG-CAA_464	0.008
	AGG-CAT-121	0.008
	ACT-CACC-207	0.010
	ACC-CAT-199	0.010

Treatment names indicate: EC: Environmental Control, HC: Historical Control, MD: Moderate Drought, SD: Severe Drought; +/-: fire/no fire.

## Discussion

Our study found very little evidences of genetic change of *C*. *ladanifer* to fire and/or drought treatments, as the experiment led to the reestablishment of populations genetically very similar to those present before fire. We found no overall effects of drought treatments on genome-wide AFLP marker diversity, no genome-wide genetic differentiation among treatments that exceeded background levels and no consistent effect on single loci. This indicates that fire and drought are unlikely to strongly affect the genetic makeup of this *C*. *ladanifer* population, in spite of reduced recruitment caused by drought. The fact that the physiological performance of seedlings in burned plots was better than that of adults in unburned plots, even under the severe drought [44], and that seedling survival did not differ between treatments, suggests that the reduced recruitment was because of reduced germination and emergence rather than higher seedling mortality [46]. The lack of genetic change suggests that reduced emergence due to drought was a random, non-selective process with no genetic imprint on *C*. *ladanifer* and no implications for the adaptive potential of this species.

### Background genetic patterns

We found that spatial genetic structure existed at the site before the onset of the experiment, although these patterns were unlikely to affect the experimental results. The small amount of genome-wide differentiation between original and additional control, unburned plots (1%), suggests a pattern of differentiation across the study site, and the variation among plots (2%) also indicates the existence of small-scale differences. The spatial analysis suggests that, at the scale of this stand, and given our sampling and set of AFLP markers, there was spatial genetic structure at all the scales investigated, i.e. within plots and among all plots. The amount of spatial genetic structure was low, with a value of the Sp statistic corresponding to outcrossing species [53]. Nevertheless, a pattern of isolation by distance was present, and indicates that gene flow cannot fully overcome the effect of genetic drift, but there is some equilibrium of gene flow and drift [59]. Restricted seed dispersal is a first candidate for this limited gene flow, since *C*. *ladanifer* has no specific dispersal mechanisms: Bastida & Talavera [60] found that only 1.6% of seeds dispersed beyond 40 cm from the canopy edge of the mother plant, although longer distance dispersal by endozoochory may occur [61]. Restricted pollen dispersal is also accountable for the pattern we found, since pollination does not seem to occur at long distances. For instance, Metcalfe & Kunin [62] found that isolated *C*. *ladanifer* plants showed no pollination success when the nearest neighbor was further than 33m. A very low pollen dispersal in *C*. *ladanifer* is also evident from the estimated pollen-to-seed dispersal ratio of 2.1 [63], which is much less than the median value of this parameter across multiple species [64]. Taken together, limited gene flow by both seed and pollen lead to some background genetic structure present at our study site, which needs to be taken into account when studying experimental effects of climate change on genetic variation.

### Effects of fire and drought on genetic differentiation

Neither fire nor drought treatments produced relevant genomic differentiation. Only a very small (0.3%), but still significant group effect was found by the hierarchical AMOVA, indicating slight differentiation among experimental treatments. However, the amount of differentiation among plots within treatments was much larger (2.6%), indicating local genetic spatial structure similar to the background pattern before the onset of the experiment. Given that the absolute level of additional experimental effect was even smaller than between original and additional EC- plots (0.82%), it is questionable whether our results, although statistically significant, can be considered biologically relevant. The pairwise hierarchical AMOVAs reinforce this suggestion, as, if any, no effects exceeding the background level (0.28%) were found. The two treatment comparisons with strongest effects were EC- vs. EC+ (0.56%), suggesting that fire can lead to differentiation. Likewise, comparison between HC+ vs. MD+ (0.61%) potentially suggests an effect of drought. However, the latter differentiation was driven by one of the HC+ plots, which was consistently differentiated (Ф_PT_>0.05) from three of the four MD+ plots. Thus, this seems to be a particular effect of plot, or plot + treatment, rather than a general treatment effect.

The outlier analyses found that only one of the markers, locus ACT-CACC_307 showed increased differentiation (F_ST_ 0.032), which was however inconsistently related to fire or drought treatments: while this marker was generally more abundant in burned plots, some of the burned plots showed low frequency, while some unburned plots showed high frequency. Only one of the genome scans identified it as an outlier, and since false positives can be common among outlier detection methods [65], this precludes drawing strong conclusions about the genetic signature of fire on our experiment. Similarly, the outliers detected by Mcheza/DFDIST were not detected by other approaches and presented very low or very high overall abundance (frequency of 3% or lower, or 96% or higher, with the exception of ACG-CAT_067 with 83%) and are likely due to random effects in individual plots and they likely are false positives. Therefore, all our analyses in combination suggest no evident genetic change in the *C*. *ladanifer* population that established after the fire, neither with respect to the previous, unburned population, nor among the different levels of drought, both with respect to genome-wide effects and to locus-specific effects.

Obligate post-fire seeder species like *C*. *ladanifer* are very drought-tolerant and show high photosynthesis and transpiration rates when water availability is high, rapid stomatal responses and high dehydration tolerance [66, 67]. Furthermore, burned areas are favorable for seedlings of *C*. *ladanifer*, and even under drought, they show better physiological status than adult plants in unburned areas [44]. Thus, the selective pressures exerted by increased drought on seedlings may not be stronger than those under normal conditions (i.e. without drought). In fact, once seedlings established, mortality decreased sharply and most plants survived in this experiment, even under extreme drought, so no significant differences were found in seedling mortality between drought and control treatments [46]. However, drought did have an effect, reducing the seedling emergence and therefore final recruitment. In our case this reduced emergence seems to be non-directional in evolutionary/genomic terms, and it is likely that germination and seedling emergence under drought may be driven by random factors such as microscale water availability in the soil, the time of emergence, etc., rather than by the variability in the response of seeds to water availability [39].

Previous studies exploring the effects of simulated climate change on plants at the genetic level have found candidate loci for selection over longer periods of time [14,15]. In the case of the Jump et al. [14] study, evidences of selection were found in *Fumana thymifolia* after drought and warming treatments. As in our study, mortality in the drought and warming treatments did not differ from the control, and the reduced recruitment in the drought treatments was due to a lower number of emergences, rather than selective mortality during the early stages of plant growth [41]. This supports our suggestion that recruitment under drought is not reduced by increased mortality of seedlings but by reduced emergence. However, since Jump et al. [14], in contrast to our study, did find potentially adaptive loci, it remains unclear whether there are species-specific differences in the germination process, whether the outlier loci they found would have been detected under other outlier detection approaches [65], or whether an adaptive response was actually taking place in our experiment but remained undetected. While the latter cannot be excluded in our study as well as in others exploring the genetic effects of climate change [22], this seems unlikely given the observed physiological and demographical response of *C*. *ladanifer* [44,46]. However, a cautionary note is appropriate here with respect to the lack of observed genetic changes. First, the experiment did not encompass the full life cycle of *C*. *ladanifer* but only the recruitment phase. Potential effects of increased drought on later phases of the life cycle on genetic variation could not be assessed, like sexual reproduction, e.g. due to phenological changes, and build-up of a seed bank, e.g. due to reduced seed production. Second, *C*. *ladanifer* is a self-incompatible species dependent on a sufficient number of nearby conspecifics for successful seed production, thus changes of plant density may affect the number of interbreeding individuals, and thus effective population size with implications for genetic variation. Third, the use of genetic markers that only target a small proportion of the genome in this non-model species limits the power to detect outlier loci.

Given that the treatments were rather extreme (drought equivalent to percentile 2 of the historical climate in annual terms), we can conclude that after an initial, random filter on germination, *C*. *ladanifer* was mostly resilient to simulated drought after fire. We found no decrease in genetic variation and therefore we do not foresee a threat to the adaptive potential of the species, since the adaptive potential of populations might associate positively with genetic diversity [10], although this interpretation of neutral diversity remains controversial [68,69]. Since this study was limited to a single population, it is uncertain whether a similar response would be found in populations across the full range of the species, which may possess different background levels of genetic variation from which selection can operate. Despite this shortcoming, this study serves as an example of how a drought-tolerant, Mediterranean keystone species can be resilient to changes in rainfall as projected under climate change, and raises the question of whether other drought-adapted, obligate seeder species in Mediterranean-type ecosystems may respond similarly to a changing climate.

## Supporting information

S1 Dataset Raw data. Presence/absence matrix of each individual allele in each sample, and sample relative coordinates.
(XLSX)

S1 Table F- and P-values from a one-way ANOVA testing fire and drought treatment effects on genetic diversity metrics of *Cistus ladanifer* L. *N*_*a*_: mean number of different alleles, *N*_*e*_: number of effective alleles, *I*: Shannon’s information index, *H*_*e*_: expected heterozygosity (assuming Hardy-Weinberg-equilibrium), *uH*_*e*_: unbiased expected heterozygosity.
(DOCX)

S2 Table Pairwise Ф_PT_ and associated P-values between plots for the AFLP locus AGG-CAT_213 in *Cistus ladanifer*. Ф_PT_ values below diagonal, P-values above diagonal. Significant values are marked in bold.
(DOCX)

S3 Table Pairwise Ф_PT_ and associated P-values between plots for the AFLP locus ACC-CTC_109 in *Cistus ladanifer*. Ф_PT_ values below diagonal, P-values above diagonal.
(DOCX)
