# Supplementary material for: No genetic adaptation of the Mediterranean keystone shrub Cistus ladanifer in response to experimental fire and extreme drought
Source: PLoS One. 2018 Jun 20;13(6):e0199119. doi: 10.1371/journal.pone.0199119 (PMC6010289; doi:10.1371/journal.pone.0199119)
Supplement: S1 Table — Na: mean number of different alleles, Ne: number of effective alleles, I: Shannon’s information index, He: expected heterozygosity (assuming Hardy-Weinberg-equilibrium), uHe: unbiased expected heterozygosity. (DOCX) [file pone.0199119.s002.docx]

**S1 Table**. **F- and P-values from a one-way ANOVA testing fire and drought treatment effects on genetic diversity metrics of *Cistus ladanifer* L.** *N_a_*: mean number of different alleles, *N_e_*: number of effective alleles, *I*: Shannon’s information index, *H_e_*: expected heterozygosity (assuming Hardy-Weinberg-equilibrium), *uH_e_*: unbiased expected heterozygosity.

|  | *F* | *P* |
| --- | --- | --- |
| *N_a_* | 1.459 | 0.252 |
| *N_e_* | 1.068 | 0.410 |
| *I* | 0.685 | 0.641 |
| *H_e_* | 0.908 | 0.497 |
| *uH_e_* | 0.920 | 0.490 |
