# Supplementary material for: No genetic adaptation of the Mediterranean keystone shrub Cistus ladanifer in response to experimental fire and extreme drought
Source: PLoS One. 2018 Jun 20;13(6):e0199119. doi: 10.1371/journal.pone.0199119 (PMC6010289; doi:10.1371/journal.pone.0199119)
Supplement: S2 Table — ФPT values below diagonal, P-values above diagonal. Significant values are marked in bold. (DOCX) [file pone.0199119.s003.docx]

**S2 Table. Pairwise Ф_PT_ and associated P-values between plots for the AFLP locus AGG-CAT_213 in *Cistus ladanifer.*** Ф_PT_ values below diagonal, P-values above diagonal. Significant values are marked in bold.

|  | EC-11 | EC-12 | EC-13 | EC-14 | EC-21 | EC-22 | EC-23 | EC-24 | EC+1 | EC+2 | EC+3 | EC+4 | HC+1 | HC+2 | HC+3 | HC+4 | MD+1 | MD+2 | MD+3 | MD+4 | SD+1 | SD+2 | SD+3 | SD+4 |
| --- | --- | --- | --- | --- | --- | --- | --- | --- | --- | --- | --- | --- | --- | --- | --- | --- | --- | --- | --- | --- | --- | --- | --- | --- |
| EC-11 |  | 0.219 | 0.217 | 0.322 | 0.373 | 0.193 | 0.375 | 0.368 | 0.194 | 0.322 | 0.380 | 0.319 | 0.148 | 0.372 | 0.365 | 0.370 | 0.324 | 0.312 | 0.367 | 0.323 | 0.213 | 0.217 | 0.313 | 0.318 |
| EC-12 | 0.000 |  | 0.479 | 0.215 | 0.308 | 0.064 | 0.153 | 0.157 | 0.069 | 0.316 | 0.314 | 0.215 | 0.311 | 0.318 | 0.160 | 0.314 | 0.218 | 0.309 | 0.322 | 0.220 | 0.482 | 0.478 | 0.578 | 0.209 |
| EC-13 | 0.000 | 0.000 |  | 0.216 | 0.312 | 0.072 | 0.162 | 0.149 | 0.071 | 0.316 | 0.318 | 0.212 | 0.312 | 0.323 | 0.158 | 0.313 | 0.212 | 0.314 | 0.317 | 0.223 | 0.469 | 0.485 | 0.591 | 0.216 |
| EC-14 | 0.000 | 0.000 | 0.000 |  | 0.155 | **0.026** | 0.375 | 0.368 | **0.027** | 0.321 | 0.159 | 0.595 | 0.361 | 0.159 | 0.375 | 0.153 | 0.597 | 0.318 | 0.155 | 0.587 | 0.216 | 0.478 | 0.310 | 0.588 |
| EC-21 | 0.000 | 0.079 | 0.079 | 0.000 |  | 0.208 | 0.398 | 0.402 | 0.216 | 0.375 | 0.194 | 0.155 | 0.408 | 0.187 | 0.389 | 0.199 | 0.159 | 0.366 | 0.188 | 0.154 | 0.309 | **0.000** | 0.371 | 0.150 |
| EC-22 | 0.124 | 0.266 | 0.266 | **0.152** | 0.000 |  | 0.404 | 0.415 | 0.229 | 0.398 | 0.218 | **0.026** | 0.413 | 0.209 | 0.420 | 0.208 | **0.028** | 0.406 | 0.211 | **0.024** | 0.065 | **0.011** | 0.406 | **0.028** |
| EC-23 | 0.039 | 0.170 | 0.170 | 0.063 | 0.000 | 0.000 |  | 0.212 | 0.404 | 0.194 | 0.402 | 0.367 | 0.403 | 0.397 | 0.218 | 0.400 | 0.375 | 0.192 | 0.401 | 0.369 | 0.155 | **0.038** | 0.401 | 0.368 |
| EC-24 | 0.039 | 0.170 | 0.170 | 0.063 | 0.000 | 0.000 | 0.000 |  | 0.412 | 0.199 | 0.397 | 0.373 | 0.401 | 0.412 | 0.210 | 0.403 | 0.368 | 0.189 | 0.401 | 0.381 | 0.153 | **0.037** | 0.411 | 0.370 |
| EC+1 | 0.124 | 0.266 | 0.266 | **0.152** | 0.000 | 0.000 | 0.000 | 0.000 |  | 0.402 | 0.213 | **0.026** | 0.415 | 0.212 | 0.412 | 0.218 | **0.027** | 0.399 | 0.220 | **0.026** | 0.067 | **0.015** | 0.398 | **0.025** |
| EC+2 | 0.000 | 0.000 | 0.000 | 0.000 | 0.000 | 0.045 | 0.000 | 0.000 | 0.045 |  | 0.371 | 0.318 | 0.374 | 0.369 | 0.195 | 0.380 | 0.319 | 0.153 | 0.374 | 0.321 | 0.315 | **0.000** | 0.370 | 0.314 |
| EC+3 | 0.000 | 0.079 | 0.079 | 0.000 | 0.000 | 0.000 | 0.000 | 0.000 | 0.000 | 0.000 |  | 0.158 | 0.397 | 0.194 | 0.400 | 0.184 | 0.160 | 0.361 | 0.198 | 0.156 | 0.317 | **0.000** | 0.373 | 0.151 |
| EC+4 | 0.000 | 0.000 | 0.000 | 0.000 | 0.000 | **0.152** | 0.063 | 0.063 | **0.152** | 0.000 | 0.000 |  | 0.373 | 0.152 | 0.361 | 0.158 | 0.591 | 0.319 | 0.157 | 0.597 | 0.212 | 0.467 | 0.322 | 0.586 |
| HC+1 | 0.000 | 0.111 | 0.111 | 0.010 | 0.000 | 0.000 | 0.000 | 0.000 | 0.000 | 0.000 | 0.000 | 0.010 |  | 0.399 | 0.397 | 0.404 | 0.370 | 0.370 | 0.408 | 0.373 | 0.308 | **0.036** | 0.360 | 0.378 |
| HC+2 | 0.000 | 0.079 | 0.079 | 0.000 | 0.000 | 0.000 | 0.000 | 0.000 | 0.000 | 0.000 | 0.000 | 0.000 | 0.000 |  | 0.400 | 0.193 | 0.151 | 0.373 | 0.191 | 0.161 | 0.318 | **0.000** | 0.367 | 0.160 |
| HC+3 | 0.039 | 0.170 | 0.170 | 0.063 | 0.000 | 0.000 | 0.000 | 0.000 | 0.000 | 0.000 | 0.000 | 0.063 | 0.000 | 0.000 |  | 0.401 | 0.376 | 0.195 | 0.403 | 0.374 | 0.151 | **0.038** | 0.400 | 0.367 |
| HC+4 | 0.000 | 0.079 | 0.079 | 0.000 | 0.000 | 0.000 | 0.000 | 0.000 | 0.000 | 0.000 | 0.000 | 0.000 | 0.000 | 0.000 | 0.000 |  | 0.158 | 0.371 | 0.193 | 0.152 | 0.319 | **0.000** | 0.371 | 0.157 |
| MD+1 | 0.000 | 0.000 | 0.000 | 0.000 | 0.000 | **0.152** | 0.063 | 0.063 | **0.152** | 0.000 | 0.000 | 0.000 | 0.010 | 0.000 | 0.063 | 0.000 |  | 0.317 | 0.157 | 0.594 | 0.217 | 0.485 | 0.309 | 0.589 |
| MD+2 | 0.000 | 0.000 | 0.000 | 0.000 | 0.000 | 0.045 | 0.000 | 0.000 | 0.045 | 0.000 | 0.000 | 0.000 | 0.000 | 0.000 | 0.000 | 0.000 | 0.000 |  | 0.373 | 0.319 | 0.317 | **0.000** | 0.377 | 0.320 |
| MD+3 | 0.000 | 0.079 | 0.079 | 0.000 | 0.000 | 0.000 | 0.000 | 0.000 | 0.000 | 0.000 | 0.000 | 0.000 | 0.000 | 0.000 | 0.000 | 0.000 | 0.000 | 0.000 |  | 0.157 | 0.312 | **0.000** | 0.374 | 0.152 |
| MD+4 | 0.000 | 0.000 | 0.000 | 0.000 | 0.000 | **0.152** | 0.063 | 0.063 | **0.152** | 0.000 | 0.000 | 0.000 | 0.010 | 0.000 | 0.063 | 0.000 | 0.000 | 0.000 | 0.000 |  | 0.218 | 0.478 | 0.321 | 0.587 |
| SD+1 | 0.000 | 0.000 | 0.000 | 0.000 | 0.079 | 0.266 | 0.170 | 0.170 | 0.266 | 0.000 | 0.079 | 0.000 | 0.111 | 0.079 | 0.170 | 0.079 | 0.000 | 0.000 | 0.079 | 0.000 |  | 0.473 | 0.590 | 0.216 |
| SD+2 | 0.111 | 0.008 | 0.008 | 0.091 | **0.273** | **0.455** | **0.364** | **0.364** | **0.455** | **0.182** | **0.273** | 0.091 | **0.314** | **0.273** | **0.364** | **0.273** | 0.091 | **0.182** | **0.273** | 0.091 | 0.008 |  | 0.096 | 0.481 |
| SD+3 | 0.000 | 0.016 | 0.016 | 0.000 | 0.000 | 0.017 | 0.000 | 0.000 | 0.017 | 0.000 | 0.000 | 0.000 | 0.000 | 0.000 | 0.000 | 0.000 | 0.000 | 0.000 | 0.000 | 0.000 | 0.016 | 0.213 |  | 0.317 |
| SD+4 | 0.000 | 0.000 | 0.000 | 0.000 | 0.000 | **0.152** | 0.063 | 0.063 | **0.152** | 0.000 | 0.000 | 0.000 | 0.010 | 0.000 | 0.063 | 0.000 | 0.000 | 0.000 | 0.000 | 0.000 | 0.000 | 0.091 | 0.000 |  |
