# Supplementary material for: No genetic adaptation of the Mediterranean keystone shrub Cistus ladanifer in response to experimental fire and extreme drought
Source: PLoS One. 2018 Jun 20;13(6):e0199119. doi: 10.1371/journal.pone.0199119 (PMC6010289; doi:10.1371/journal.pone.0199119)
Supplement: S3 Table — ФPT values below diagonal, P-values above diagonal. (DOCX) [file pone.0199119.s004.docx]

**S3 Table. Pairwise Ф_PT_ and associated P-values between plots for the AFLP locus ACC-CTC_109 in *Cistus ladanifer.*** Ф_PT_ values below diagonal, P-values above diagonal.

|  | EC-11 | EC-12 | EC-13 | EC-14 | EC-21 | EC-22 | EC-23 | EC-24 | EC+1 | EC+2 | EC+3 | EC+4 | HC+1 | HC+2 | HC+3 | HC+4 | MD+1 | MD+2 | MD+3 | MD+4 | SD+1 | SD+2 | SD+3 | SD+4 |
| --- | --- | --- | --- | --- | --- | --- | --- | --- | --- | --- | --- | --- | --- | --- | --- | --- | --- | --- | --- | --- | --- | --- | --- | --- |
| EC-11 |  | - | - | - | 0.483 | - | 0.478 | - | - | - | - | - | - | - | - | - | - | - | - | - | 0.480 | 0.472 | - | 0.477 |
| EC-12 | - |  | - | - | 0.478 | - | 0.477 | - | - | - | - | - | - | - | - | - | - | - | - | - | 0.474 | 0.475 | - | 0.473 |
| EC-13 | - | - |  | - | 0.482 | - | 0.482 | - | - | - | - | - | - | - | - | - | - | - | - | - | 0.475 | 0.472 | - | 0.472 |
| EC-14 | - | - | - |  | 1.000 | - | 1.000 | - | - | - | - | - | - | - | - | - | - | - | - | - | 0.214 | 0.479 | - | 1.000 |
| EC-21 | 0.000 | 0.000 | 0.000 | 0.000 |  | 1.000 | 0.477 | 1.000 | 1.000 | 1.000 | 1.000 | 1.000 | 0.480 | 1.000 | 1.000 | 1.000 | 1.000 | 1.000 | 1.000 | 1.000 | 0.221 | 0.221 | 0.481 | 0.471 |
| EC-22 | - | - | - | - | 0.000 |  | 1.000 | - | - | - | - | - | - | - | - | - | - | - | - | - | 0.214 | 0.478 | - | 1.000 |
| EC-23 | 0.000 | 0.000 | 0.000 | 0.000 | 0.000 | 0.000 |  | 1.000 | 1.000 | 1.000 | 1.000 | 1.000 | 0.477 | 1.000 | 1.000 | 1.000 | 1.000 | 1.000 | 1.000 | 1.000 | 0.219 | 0.222 | 0.480 | 0.475 |
| EC-24 | - | - | - | - | 0.000 | - | 0.000 |  | - | - | - | - | - | - | - | - | - | - | - | - | 0.215 | 0.483 | - | 1.000 |
| EC+1 | - | - | - | - | 0.000 | - | 0.000 | - |  | - | - | - | - | - | - | - | - | - | - | - | 0.213 | 0.485 | - | 1.000 |
| EC+2 | - | - | - | - | 0.000 | - | 0.000 | - | - |  | - | - | - | - | - | - | - | - | - | - | 0.221 | 0.470 | - | 1.000 |
| EC+3 | - | - | - | - | 0.000 | - | 0.000 | - | - | - |  | - | - | - | - | - | - | - | - | - | 0.216 | 0.482 | - | 1.000 |
| EC+4 | - | - | - | - | 0.000 | - | 0.000 | - | - | - | - |  | - | - | - | - | - | - | - | - | 0.215 | 0.486 | - | 1.000 |
| HC+1 | - | - | - | - | 0.000 | - | 0.000 | - | - | - | - | - |  | - | - | - | - | - | - | - | 0.470 | 0.476 | - | 0.478 |
| HC+2 | - | - | - | - | 0.000 | - | 0.000 | - | - | - | - | - | - |  | - | - | - | - | - | - | 0.217 | 0.475 | - | 1.000 |
| HC+3 | - | - | - | - | 0.000 | - | 0.000 | - | - | - | - | - | - | - |  | - | - | - | - | - | 0.218 | 0.482 | - | 1.000 |
| HC+4 | - | - | - | - | 0.000 | - | 0.000 | - | - | - | - | - | - | - | - |  | - | - | - | - | 0.222 | 0.475 | - | 1.000 |
| MD+1 | - | - | - | - | 0.000 | - | 0.000 | - | - | - | - | - | - | - | - | - |  | - | - | - | 0.221 | 0.484 | - | 1.000 |
| MD+2 | - | - | - | - | 0.000 | - | 0.000 | - | - | - | - | - | - | - | - | - | - |  | - | - | 0.220 | 0.482 | - | 1.000 |
| MD+3 | - | - | - | - | 0.000 | - | 0.000 | - | - | - | - | - | - | - | - | - | - | - |  | - | 0.219 | 0.483 | - | 1.000 |
| MD+4 | - | - | - | - | 0.000 | - | 0.000 | - | - | - | - | - | - | - | - | - | - | - | - |  | 0.214 | 0.484 | - | 1.000 |
| SD+1 | 0.100 | 0.100 | 0.100 | 0.111 | 0.000 | 0.111 | 0.000 | 0.111 | 0.111 | 0.111 | 0.111 | 0.111 | 0.100 | 0.111 | 0.111 | 0.111 | 0.111 | 0.111 | 0.111 | 0.111 |  | 0.311 | 0.477 | 0.220 |
| SD+2 | 0.081 | 0.081 | 0.081 | 0.091 | 0.000 | 0.091 | 0.000 | 0.091 | 0.091 | 0.091 | 0.091 | 0.091 | 0.081 | 0.091 | 0.091 | 0.091 | 0.091 | 0.091 | 0.091 | 0.091 | 0.000 |  | 0.471 | 0.218 |
| SD+3 | - | - | - | - | 0.000 | - | 0.000 | - | - | - | - | - | - | - | - | - | - | - | - | - | 0.100 | 0.081 |  | 0.475 |
| SD+4 | 0.000 | 0.000 | 0.000 | 0.000 | 0.000 | 0.000 | 0.000 | 0.000 | 0.000 | 0.000 | 0.000 | 0.000 | 0.000 | 0.000 | 0.000 | 0.000 | 0.000 | 0.000 | 0.000 | 0.000 | 0.000 | 0.000 | 0.000 |  |
